# Diet Quality and Past Changes in Food Consumption as Determinants of Intentions to Eat Less Meat and More Plant-Based Foods

**DOI:** 10.3390/foods13233767

**Published:** 2024-11-24

**Authors:** Marzena Jeżewska-Zychowicz, Marta Sajdakowska, Jerzy Gębski, Małgorzata Kosicka-Gębska, Krystyna Gutkowska

**Affiliations:** Institute of Human Nutrition Sciences, Warsaw University of Life Sciences (SGGW-WULS), 02-776 Warsaw, Poland; marzena_jezewska_zychowicz@sggw.edu.pl (M.J.-Z.); jerzy_gebski@sggw.edu.pl (J.G.); malgorzata_kosicka_gebska@sggw.edu.pl (M.K.-G.); krystyna_gutkowska@sggw.edu.pl (K.G.)

**Keywords:** diet quality, food intake, adults, cross-sectional study

## Abstract

This study aimed to examine the relationship between self-reported changes in food consumption over the past 2 years, quality of actual diet, and declared intentions to eat more plant-based foods while reducing meat consumption in the next year. A cross-sectional study using Computer-Assisted Web Interview (CAWI) was conducted on 1003 Polish adults in 2023. The Beliefs and Eating Habits Questionnaire (KomPAN) was used to assess the frequency of consuming various food groups and to calculate diet quality indices. Four distinct segments (“No change”, “All products limited”, “Changes in meat”, and “Less red meat, more other products”) were identified using cluster analysis. Logistic regression analysis verified the associations between these clusters, diet quality indices, and intended changes in plant-based food and meat consumption. The study results showed that most pre-existing changes involved reduced red meat intake (52%). The “No change” cluster (47.9%) was represented by more men, people with lower education, and those with a lower overall dietary quality index (DQI). A higher quality diet (as indicated by the DQI) was associated with a greater tendency to increase plant food consumption and reduce meat consumption across the entire group and within each cluster. A better understanding of how changes in food consumption relate to diet quality and intended changes in plant-based food and meat consumption can inform interventions promoting sustainable consumption, considering both nutritional recommendations and environmental requirements.

## 1. Introduction

The consumption of animal-based foods has been linked to detrimental environmental impacts [1,2]. Specifically, the production of such foods generates high amounts of greenhouse gas emissions, requires extensive land use, and can contribute to water scarcity [3,4] However, plant-based alternatives to animal products offered on the market are in many cases highly processed products [5]; thus their production requires high energy usage [6] which negatively affect the environment as well. However, this impact is lower compared to the production of animal products [7,8].

Moreover, extensive consumption of red and processed meats is associated with an increased risk of several chronic diseases [9], including, among others, cardiovascular diseases [10], cancer [11,12], type 2 diabetes [13], digestive system diseases [14,15], as well as neurodegenerative disorders and mental illnesses such as depression or anorexia [16]. This risk is further elevated by poor dietary habits, overweight, smoking, and insufficient physical activity [17]. Therefore, public health strategies should focus on improving energy balance and shifting toward a diet with less meat and more plant-based foods, which would benefit both global health and the environment [18].

In line with this goal, dietary guidelines in many European countries recommend reducing red and processed meat intake [19]. Instead, a diet comprising whole grains, vegetables, fruits, legumes, nuts, unsaturated oils, and small to moderate amounts of seafood and poultry is recommended [2]. In response to these recommendations, diets with less meat and more plant-based products are gaining popularity in some countries, including the “flexitarian” diet, which primarily involves eating plant foods while occasionally including meat [20,21,22].

Switching from animal to plant-based food is important from a food security and sustainability perspective [23] and for health reasons [24,25]. However, replacing animal- with plant-based proteins may require a “profound social transformation” [26]. However, the high importance of meat in many cultures makes it challenging to replace meat with plant-based protein products [27,28]. Traditionally, meat was mainly eaten during festive occasions, but meat eating has now become a daily practice [29]. Many people associate meat with abundance and a nutritious diet [30]. Polish cuisine is traditionally focused on meat, which can lead to challenges for vegans and vegetarians, resulting in a lack of acceptance from others [31]. Consumers may also have concerns about the healthiness and naturalness of processed plant protein products [32,33]. Individuals differ in their motives for eating meat, which influences their behavior. For example, concerns about animal welfare predict lower meat intake, whereas weight-loss motives are associated with higher meat consumption [34]. However, consumers’ preferences for plant-based foods are constantly increasing due to interests in sustainability, health, and animal welfare [35,36].

Despite much research on the shift from animal-based to plant-based foods, it still needs to continue. More research attention has focused on the motivation for such a shift [32,33,35]. Little is known about the relationship between the quality of the current diet and the willingness to change it to increase consumption of plant-based foods. In addition, few studies show this change as a process, as this requires longitudinal studies, which are rarely undertaken due to the necessary commitment. Our study treats change as a process (past–present–future), with the stages of the process being presented based on the self-perceptions of the study participants. Moreover, previous studies usually do not account for differences in the quality of plant foods, which affects health outcomes. Higher intake of healthy plant foods, such as whole grains, vegetables, fruits, and nuts, is associated with lower all-cause mortality [24,37] and favorable weight outcomes [38]. In contrast, higher intakes of less healthy plant foods, such as potatoes and sugar, are associated with a higher risk of cardiometabolic and other diseases [24,25]. Therefore, in our research alongside this dichotomous approach, i.e., plant versus meat, a more nuanced approach was introduced concerning both changes in dietary intake and diet quality by considering specific selected plant foods.

As a plant-based food, legumes offer greater sustainability because they are less harmful to the environment than animal-derived foods [39]. They are also more cost-effective than animal proteins. However, legume proteins are still considered inferior to animal proteins [40]. Despite their high protein content, legumes have lower digestibility than cereal proteins. To address this disadvantage, legumes are often combined with cereals to enhance their protein quality [41]. Despite these shortcomings, legumes contribute to a beneficial nutrient intake for adults and children and support healthier body weight [42]. They can also help achieve current environmental goals, such as reducing animal production and consumption of animal-derived foods.

The transition from animal-based to plant-based foods is the subject of much research and debate due to the difficulty in changing eating habits, a central aspect of people’s lifestyles [43] and sociocultural environments [44]. The perception that a plant-based diet is inconvenient requires excessive time and skill for food preparation and involves costly ingredients, making it challenging to reduce meat consumption [45]. The difficulties identified in modifying diets suggest the need for more flexible approaches. Increasing the amount of plant foods in your diet without eliminating animal products makes it easier to make the change because it allows for gradual changes in your diet. Cutting out all animal products is unnecessary to obtain health benefits, as moderate consumption of animal products such as fish, poultry, and dairy has been shown to have health benefits [46]. Changes in food consumption can have both positive and negative effects. One negative outcome of changing eating behaviors is a decline in diet quality [47], which may reduce quality of life [48] and contribute to deteriorating health [49,50].

Thus, this study aimed to examine self-reported changes in food consumption over the last 2 years in Poland, the relationship between these changes and the quality of actual diets, and the intentions to consume more plant-based foods and less meat in the coming year. First, we aimed to identify clusters based on self-reported changes in meat consumption (red and white), milk and dairy products, and legume products before the study. Second, we examined the characteristics of these clusters in terms of sociodemographic factors, actual diet quality, and intended changes for the following year.

## 2. Materials and Methods

### 2.1. Study Design and Sample Collection

The data were collected in Poland between June and September 2023 using a Computer-Assisted Web Interview technique as a cross-sectional study. Detailed information about sample collection is presented in a previously published paper [51]. The study was approved by the Ethics Committee of the Warsaw University of Life Sciences in Poland (Resolution No. 8/RKE//2023/U) and conducted in agreement with the guidelines of the Declaration of Helsinki. The study sample comprised 1003 Polish adults.

### 2.2. Dietary Data and Sociodemographic Data

To calculate the diet quality indices, questions on the frequency of consumption of 24 food groups from the Beliefs and Eating Habits Questionnaire (KomPAN) were used [52]. KomPAN has been validated for Polish adults [53]. Participants reported their habitual frequency of eating these foods over the 3 months preceding the survey using one of the following responses: 1—less than once a month or never; 2—1–3 times a month; 3—once a week; 4—a few times a week; 5—once a day; and 6—a few times a day. During data analysis, these responses were converted to reflect the daily frequency of intake as follows: 0—less than once a month or never; 0.06—1–3 times a month; 0.14—once a week; 0.5—a few times a week; 1—once a day; and 2—a few times a day [54]. Based on these data, three diet quality indices were calculated:

– The Prohealthy Diet Index (pHDI) is calculated based on the frequency of 10 food groups with a potentially positive effect on health, i.e., wholemeal bread, wholemeal bread rolls, buckwheat, oats, wholegrain pasta, or other coarse-ground groats; milk; fermented milk beverages; fresh cheese curd products; white meat; fish; pulse-based foods; fruit; vegetables.
– The Non-healthy Diet Index is calculated based on 14 food groups with a potentially harmful effect on health, i.e., white bread and bakery products; white rice, white pasta, fine ground groats; fast foods; fried foods; butter; lard; cheese; cold meats, smoked sausages, hot dogs; red meat; sweets, tinned meats, sweetened carbonated beverages; energy drinks; alcoholic beverages.
– The Diet-Quality Index (DQI) was calculated based on the consumption of 24 food groups, including 10 groups with a potentially positive effect on health (pHDI) and 14 groups with a potentially negative effect on health (nHDI) [54].

According to the procedure for calculating the DQI, pHDI, and nHDI, the daily frequency of intake was calculated separately for both food groups, i.e., pHDI and nHDI. The results for pHDI were multiplied by 100/20, and the results for nHDI were multiplied by 100/28. Finally, the two results were added together. The DQI ranged from −100 to 100 points. A range from −100 to −26 indicated a high intensity of non-healthy dietary characteristics, a score from −25 to 25 indicated a low intensity of non-healthy and healthy dietary characteristics, and a score from 26 to 100 showed a high intensity of healthy dietary characteristics [54]. 

Changes in food intake over the 2 years preceding the study were examined using the question: “How do you assess the changes in the consumption of four food groups over the last 2 years?” The food groups were defined as follows: (1) red meat, including pork, beef, veal, mutton, lamb, and game, in the form of dishes or ready-made products such as cold cuts and sausages; (2) white meat, including chicken, turkey, and rabbit, in the form of meals or ready-made products such as cold cuts and sausages; (3) milk and dairy products, including lactose-free products; and (4) legumes, including beans, peas, soybeans, and lentils. The nature of the changes was categorized as: 1—“I have not consumed these products”; 2—“Consumption has decreased”; 3—“Consumption has not changed”; and 4—“Consumption increased”. 

Respondents also declared their intention to change their consumption of plant-based foods and meat and meat products. The questions were: “Do you intend to eat more plant-based food in the upcoming year?” (yes/no) and “Do you intend to eat less meat and meat products in the upcoming year?” (yes/no). 

In the last part of the questionnaire were questions on sociodemographic characteristics, including age (in years), place of residence, education, and self-reported financial situation. 

## 3. Statistical Analysis

The sociodemographic characteristics of the study sample were obtained using descriptive statistics. Data were reported as percentages (%) for categorical data or as means and standard deviations (SDs) for continuous data. The normality of the distribution of continuous variables was assessed using the Kolmogorov–Smirnov test. The independence χ^2^ test assessed differences between groups based on sociodemographic characteristics. Correspondence analysis was used to present changes in the consumption of the analyzed products across clusters. Student’s *t*-test was used to determine differences between clusters. The level of statistical significance was set at *p* < 0.05.

The segmentation of respondents was conducted in a two-step process. The variables used for segmentation were recoded as follows: those who declared a decrease in consumption were assigned a value of (−1); those who declared no change or no consumption of a given product group were assigned a value of (0); and those who reported an increase in consumption were assigned a value of (1). The first stage involved cluster analysis using hierarchical methods. The second stage employed the nonhierarchical k-means method, with initial cluster seeds derived from the hierarchical method [55]. Four clusters were obtained as a result of the analysis. The accuracy of the cluster separation was assessed using the Cubic Clustering Criteria (CCC) and the pseudo-F statistic, both of which confirmed that the segments were correctly separated. The identified segments were profiled in terms of sociodemographic variables (gender, education, age) and diet quality indicators (nHDI, pHDI, DQI). Logistic regression models were used to assess the association between diet quality indices and the declarations of decreased meat consumption or increased consumption of plant-based products.

Statistical analysis was conducted using IBM SPSS Statistics for Windows, version 27.0 (IBM Corp, Armonk, NY, USA), and the SAS 9.4 statistical package (SAS Institute, Cary, NC, USA).

## 4. Results

### 4.1. Description of the Study Sample 

Table 1 presents the sociodemographic characteristics of the study sample. The sample consisted of 1003 adults aged 18 to 83 years.

### 4.2. Changes in Food Consumption 

Changes in food consumption over the 2 years preceding the study are presented in Table 2. Approximately 3/5 of people did not change their consumption of white meat (63.5%), milk and dairy products (63.2%), and dishes made from legumes (60.9%) during this period. The smallest proportion of respondents did not change their consumption of red meat (48.0%). In comparison, nearly twice as many reported a reduction in red meat consumption (42.5%) compared to other food groups (22.7%, 19.0%, and 19.8%, respectively). Only 4.1% of people increased their consumption of red meat, whereas a higher proportion increased their consumption of milk and dairy products (14.1%).

Approximately 3/5 of respondents (60.4%) declared an intention to eat more plant-based foods in the coming year, while 42.2% indicated a willingness to reduce their meat consumption over the next 12 months.

### 4.3. Diet Quality 

The mean value of DQI was 4.0 points (SD = 11.85), ranging from −34.2 to 50.9 points. Only 8 respondents (0.8%) had diets with a harmful effect on their health, characterized by a high intensity of non-healthy dietary characteristics (DQI lower than −25 points). In contrast, 48 respondents (4.8%) had diets that were beneficial to their health, marked by a high intensity of healthy dietary characteristics (DQI higher than 25 points). 

The mean value of pHDI was 23.1 points (SD = 12.14), ranging from 2.4 to 78.2 points. Only seven respondents (0.7%) exhibited a high intensity of prohealthy dietary characteristics (pHDI higher than 66 points), while 151 respondents (15.1%) showed a medium intensity of prohealthy dietary characteristics (pHDI between 33 and 66 points). The mean value of nHDI was 19.2 points (SD = 10.25), ranging from 2.4 to 78.6 points. Only 3 respondents (0.3%) had a high intensity of non-healthy dietary characteristics (nHDI higher than 66 points), while 80 respondents (8.0%) exhibited a medium intensity of non-healthy dietary characteristics (nHDI between 33 and 66 points).

### 4.4. Characteristics of the Clusters

Table 3 presents the sociodemographic characteristics of the identified clusters. Significant differences were observed only in gender, education, and age (in years).

The “No change” cluster was predominantly male and people with primary education. In the “All products limited” cluster, there were more women than men, and respondents were more likely to have vocational or higher education. The “Changes in meat” cluster primarily comprised women and people with secondary or higher education. The “Less red meat, more other products” cluster had a higher proportion of women and a greater number of people with secondary education than the other clusters. This cluster also differed significantly in age, with its members being the youngest compared to those in other clusters. The remaining clusters did not show significant age differences.

**Figure 1:**
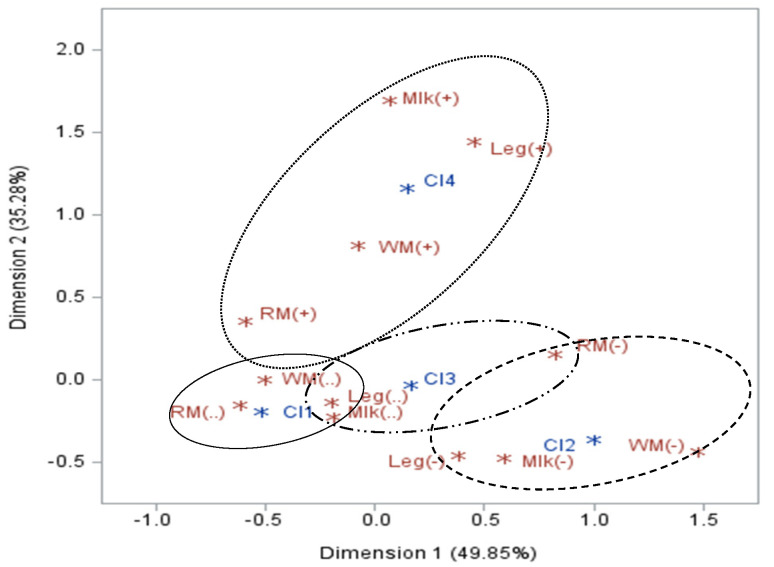
Multiple Correspondence Analysis of changes in consumption of analyzed products across clusters. RM—red meat; WM—white meat; MLK—milk and dairy products; LEG—legumes; (-)—decrease; (..)—no change; (+)—increase. Cl1–4—clusters. *—point on the graph.

The assessment of changes in the level of consumption of the analyzed products relative to cluster membership was visualized using Multiple Correspondence Analysis (MCA) (Figure 1). The two-dimensional figure explained 85.13% of the variation. In the figure, indices for all analyzed products indicating no change are located close to cluster 1, labeled “No change”. Cluster 2, labeled “All products limited”, includes the indices for all products with a reduction in consumption. Cluster 3, labeled “Changes in meat”, shows indices for all products indicating no change in consumption except for red meat, whose index indicating a reduction in consumption is positioned on the positive side of Dimension 1. Cluster 4, labeled “Less red meat, more other”, contains indices for all products with an indication of increased consumption except for red meat, which, despite a positive change in consumption, is positioned on the negative side of Dimension 1. 

Characteristics of the identified clusters, including diet quality indices, are presented in Table 4.

The highest nHDIs were recorded in the “No change” and “Less red meat, more other products” clusters, and were significantly higher than in the other two clusters. The “All products limited” cluster had the lowest nHDI, significantly lower than the other clusters. The highest pHDI was observed in the “Less red meat, more other products” cluster, and was significantly higher than in the other clusters. The lowest pHDI was found in the “No change” and “All products limited” clusters, with no significant difference between these two but they were significantly lower than in the other clusters. The “Meat changes” cluster had a significantly higher pHDI than the “All products limited” cluster. The lowest DQI was recorded in the “No change” cluster, and was significantly lower than in the other clusters. The remaining clusters did not differ significantly in terms of DQI.

### 4.5. Identified Clusters, Diet Quality, and Declared Changes

The results of the logistic regressions are presented in Table 5. Respondents who declared that they would eat more plant-based foods and less meat next year were more likely to have higher pHDI (OR: 1.23, 95% CI 1.16; 1.31 and OR: 1.11, 95% CI 1.05; 1.17, respectively) and higher DQI (OR: 1.05, 95% CI 1.04; 1.06 and OR: 1.05, 95% CI 1.03; 1.06, respectively). Respondents who indicated they would eat less meat and meat products next year were more likely to have a lower nHDI (OR: 0.90, 95% CI 0.86; 0.95).

In all clusters, people who declared their intention to eat more plant-based foods and less meat in the year following the study were more likely to have a higher DQI than those who did not. The intention to eat more plant-based foods increased from 3% in the “No change” cluster to 7% in the “All products limited” cluster with each 1-point increase in DQI. The intention to eat less meat increased by 3–4% with a 1-point increase in DQI. Across all clusters, people who intended to eat more plant-based foods in the upcoming 12 months were more likely to have a higher pHDI than those who did not declare such an intention. The intention to eat more plant-based foods increased from 18% and 19% in the “Changes in meat” and “No change” clusters, respectively, and to 24% and 27% in the “Less red meat, more other products” and “All products limited” clusters, respectively, with each 1-point increase in DQI. Only in the “No change” and “All products limited” clusters were people who intended to eat less meat next year more likely to have a higher pHDI (by 10% and 17%, respectively) compared to those who did not declare such an intention. In the “All products limited” cluster, people who intended to eat more plant-based foods were less likely to have a higher nHDI (by 12% with each 1-point increase in nHDI). In the “Changes in meat” and “Less red meat, more other products” clusters, people who intended to eat less meat were less likely to have a higher nHDI (by 13% with each 1-point increase in nHDI).

## 5. Discussion

The study aimed to examine self-reported changes in food consumption over the previous 2 years among Polish adults and to explore the relationship between these changes, the quality of actual diet, and intentions to eat more plant-based foods and less meat in the coming year. Four specific consumer clusters were identified based on self-reported changes in meat consumption (red and white meat), milk and dairy products, and legume products. The characteristics of these clusters considered sociodemographic factors, actual diet quality, and intended changes in the consumption of plant-based foods and meat in the following year. 

The results show that food-consumption patterns were relatively stable, with about 3/5 of respondents not changing their consumption of white meat, milk, and dairy products, and legumes over the 2 years preceding the study. Approximately half of the sample changed their consumption of red meat, with most changes involving a reduction in consumption. These results confirm that eating habits change slowly [43], which may be significantly influenced by the sociocultural environment [44]. Nevertheless, the 2-year period covered by the declared changes was relatively short from the perspective of the change process. This period also followed the pandemic, during which significant changes in food consumption were observed both in Poland [56,57] and in other countries [58,59,60]. Factors influencing these changes included pandemic-related restrictions, changes in grocery shopping frequency, income losses due to the pandemic, sociodemographic characteristics, and individuals’ perceived risk of COVID-19 [61]. The situation during the pandemic may have contributed to the stabilization of consumption immediately after its end. 

Despite the relatively high stability in the consumption of selected foods and the fact that meat consumption is a strongly habitual behavior [62], a substantial percentage of the sample reduced their consumption of red meat. This reduction aligns with changes observed in other countries [63,64,65] and is consistent with statistical data on meat consumption and dietary recommendations [2,19]. In Poland, in 2022, there was a decrease in beef consumption, stable pork consumption, and an increase in poultry consumption compared to 2000 [66]. These trends have been stable since 1981, when a significant decline in beef consumption was observed, especially due to its high price relative to its quality and other kinds of meat [67]. In addition to self-reported changes in red meat consumption, most respondents indicated a willingness to reduce meat consumption further in the future, which may confirm the stability of previously made changes. From the perspective of desired pro-environmental activities, it is also notable that approximately 3/5 of respondents declared their intention to eat more plant-based foods. Therefore, the results confirm the observed trends in the preferences and consumption of plant-based and animal foods [35,36].

More women than men have changed their consumption of meat, dairy foods, and legumes, while more men have not altered their food intake over the past 2 years. A significant barrier to changes in meat consumption among men may be that they consume more eggs, milk products, fish, and meat than women [68,69,70]. Moreover, men tend to eat much more red and processed meat [71] and report a higher preference for meat than women [72,73]. Nevertheless, while gender differences have been primarily observed regarding food intake or dietary patterns, less is known about gender differences in dietary changes [74]. However, ref. [75] observed that in a group of people over 55, men made favorable dietary changes by increasing vegetable intake and reducing salt, sugar, and alcohol consumption. In addition, those who changed their diet over the past 2 years were differentiated by education level. More participants with higher education reported reducing their consumption of all products, while others only changed meat consumption. More people with secondary education modified their meat consumption and represented the largest proportion of people in the “Red meat limited and other products increased” cluster. This cluster also had the highest number of the youngest people. The variation in declared changes based on education may result from the differing impact of education on meat consumption [76], which varies between countries [77]. The reduction in red meat consumption among the youngest group (the “Less red meat, more other products” cluster) may be linked to the transition into adulthood, including leaving the family home [62], as well as their greater tendency to adopt healthier and more environmentally sustainable eating patterns [78]. The lack of age-related differences in the other clusters could be attributed to the fact that age itself is not considered an important predictor of meat consumption. However, age may play a role in determining gender differences in meat consumption, influenced by the development of biological differences, which reach a maximum between the ages of 51 and 65 years [79]. Older consumers, being more traditional and less inclined to change their behavior, were expected to show greater differences between clusters in terms of reported changes; however, this was not confirmed by the study’s results. 

People who reported reducing their meat consumption over the previous 2 years exhibited different patterns of change. Among them, some reduced their intake of both red and white meat (“All products limited” cluster: 19.5%). Others primarily reduced their consumption of red meat, with white meat intake either remaining unchanged (79.9%) or even increasing (20.1%) (“Changes in meat” cluster: 18.3%). Additionally, some people reduced their intake of red meat only (“Less red meat, more other products” cluster: 14.3%). In the latter two cases, the reduction in red meat consumption, combined with either increased or unchanged white meat consumption, may be connected to a greater attachment to the cultural significance of meat [27,28]. Decreases, no changes, or increases in the consumption of other products also accompanied decreases in meat consumption. The co-occurrence of various changes in food intake needs to be monitored due to their potential impact on diet quality, including the risk of deterioration [34,80].

The identification of the “Changes in meat” cluster confirmed a trend observed in previous studies, where poultry was the main driver of increasing total meat consumption. In contrast, the consumption of beef and sheep meat has generally decreased [22,81]. The main reasons for these observed downturns in consumption are related to the economic situation, sustainability or health concerns, and individual factors [82,83]. Campaigns conducted in recent years against the consumption of red meat may also play a significant role [2]. In addition, the changes implemented by respondents in the “All products limited” cluster may partially reflect modifications initiated during the pandemic period. Previous studies have shown that during lockdown, people in many countries shopped less frequently, leading to a general decline in the consumption of fresh foods, especially fruits and vegetables, as well as meat [61,84]. In addition, the overall reduction in food intake could be linked to the widespread adoption of dietary restrictions [85].

The study’s results confirmed the stability of plant food and meat consumption but also reflected an approach indicating gradual changes towards a healthier plant-based diet without excluding entire food groups (e.g., certain animal foods) [80]. Almost half of the respondents declared stability in their consumption of the foods included in this study (“No change” cluster: 47.9%). Although food choices are recognized as dynamic and evolving throughout life [86], they are also considered relatively stable over shorter periods [75]. Major shifts in food choice patterns are initiated by significant life events such as leaving school, changing employment, or changing personal relationships [87], which may have been limited in these 2 years. The lack of change in meat consumption may be attributed to the habitual nature of eating these products and their cultural significance [88,89]. The observed diversity in changes in the consumption of animal-based foods and legumes may partially support an approach suggesting different pathways to a more plant-based diet, namely sustainability by stealth, moderate engagement, and cultural change [21]. Nevertheless, further studies are needed to extend the observation period and deepen the knowledge of consumer motivations driving these changes.

Despite the short period over which changes in food consumption were reported, they have shown a relationship with overall diet quality (DQI). DQI showed no differences among the three clusters where food consumption was modified. At the same time, DQI was significantly higher in those clusters than the “No change” cluster, indicating better diet quality in cases where changes were made over the previous 2 years. The nature of the study, however, does not allow for establishing a causal relationship between these changes and the current DQI. Thus, there is a need to monitor changes in food consumption using both subjective and objective indicators in longitudinal studies to assess their cause-and-effect relationship. Nevertheless, previous studies have already confirmed that changes in the intake of foods such as legumes, vegetables, and dairy affect overall diet quality [90,91]. The better diet quality (higher DQI) among those who have made changes can be explained in two ways: either as a result of the changes made [90] or because people who eat more healthily are more likely to follow dietary and lifestyle recommendations and therefore more likely to make changes [75,92]. 

Diet quality within the clusters, in addition to the differences above in DQI, varied in terms of specific characteristics, as evidenced by the pHDI and nHDI indices. The highest nHDI was observed in the “Less red meat, more other products” cluster, while the “All products limited” cluster had the lowest nHDI and also recorded a low pHDI. Thus, a limited food intake, including a reduction in red meat consumption, was associated with better diet quality, resulting from the reduction of both negative dietary characteristics (low nHDI) and positive dietary characteristics (low pHDI). In contrast, no change in dietary intake (the “No change” cluster) was associated with the poorest dietary quality, i.e., the lowest DQI and pHDI, with pHDI not differing from that observed in the “All products limited” cluster. In addition, the “No change” and “Less red meat, more other products” clusters recorded the highest nHDI. Thus, the overall diet quality was significantly better when any change in the food groups’ consumption occurred over the previous 2 years. Differences in nHDI and pHDI were associated with the nature of the change, with dietary restrictions linked to lower nHDI. At the same time, variations in meat intake and increases in nonmeat intake favored a higher pHDI. However, the interpretation of these results is limited by the lack of evaluation of diet quality indices from 2 years before the study due to its cross-sectional nature. Longitudinal studies, however, show that positive changes in food consumption improve diet quality over time, as confirmed by overall dietary and health-promoting indices [75,80,90,93]. Future studies should include indices of diet quality that separately assess its healthy and unhealthy aspects. This approach is supported by the results for the “Less red meat, more other products” cluster, where a decrease in red meat and an increase in other products, including legumes, were observed. In this case, the higher pHDI can be attributed to the nutritional characteristics of milk, dairy products, and legumes as representatives of plant foods. Conversely, the high nHDI in this cluster can be explained by including other product groups, such as fast food and sweets, in this indicator. Increased consumption of these products may have contributed to the deterioration of the nHDI. Nevertheless, the improvement in pHDI was greater than the decline in nHDI, resulting in an overall increase in DQI.

This study’s results confirm that the current diet’s quality is associated with intended changes across the group, indicating its usefulness in predicting and promoting beneficial changes in plant-based and meat-based food consumption. Specifically, a higher quality diet (DQI) was linked to a greater tendency to increase plant food consumption and reduce meat consumption. Higher pHDI was associated with a greater tendency to increase plant food consumption in all clusters and to decrease meat consumption, but only among those reducing their intake of all products or those making no change. Consuming fewer unhealthy foods (lower nHDI), which improves DQI, favored declarations of meat reduction in a group of people who had primarily limited their consumption of red meat. In addition, lower nHDI favored increased plant-based food intake among those who had previously limited their intake of all products. Thus, increasing plant food intake may help counter deficiencies resulting from reducing so many food groups. The results indicate that people with diets characterized by low overall quality, a low proportion of health-promoting foods, or a high proportion of unhealthy foods did not declare changes that could improve their diet quality [34,80,94], which may contribute to increased disease and mortality [95]. The study revealed differences in how previous and intended changes in the consumption of plant and animal foods related to health-promoting (pHDI) and non-health-promoting (nHDI) dietary indices compared to the overall dietary index (DQI). Additionally, the contribution of previously implemented changes to predicting future food consumption varied, suggesting that further research should employ more detailed dietary indicators beyond general dietary indices. 

## 6. Conclusions

The study results allow us to conclude that a tendency to change consumption patterns regarding animal-based foods and legumes occurs among about half of the sample. A higher tendency to change food consumption over the previous 2 years was particularly noted among women, younger people, and those with a higher level of education. The overall diet quality was significantly higher when any change in the consumption of the considered food groups was introduced over the previous 2 years. Variation in nHDI and pHDI across clusters indicated an association with the nature of the change: reductions in food intake were associated with lower nHDI, while modifications in meat intake and increases in nonmeat intake promoted higher pHDI. Superior diet quality (DQI) was associated with a greater tendency to increase plant food consumption and reduce meat consumption across the entire group. Differences were observed in the associations of previous and intended changes in the consumption animal-based foods with health-promoting (pHDI) and non-health-promoting (nHDI) dietary indices compared to the overall dietary index (DQI). Additionally, the contribution of previously implemented changes to predicting future plant food and meat consumption differed. A better understanding of how previous changes in food consumption predict future changes in plant food and meat consumption and their relationship with diet quality can serve as the basis for interventions promoting sustainable consumption, considering both nutritional recommendations and environmental requirements.

## 7. Limitations of the Study

This study has certain limitations related to survey data, which may contribute to some bias. One limitation is that the study relied on self-reported information, which may result in measurement error. We used the FFQ to collect dietary data, and it has been shown that self-reported dietary intake data are often skewed due to some degree of misreporting, including underestimation and overestimation of nutrient intakes [96]. Overestimation of the frequency of consuming health-promoting foods and underestimation of unhealthy foods may have affected the calculated diet quality indices. The cross-sectional design of this study also limits the interpretation of the results, as it does not allow for establishing a causal relationship between diet quality and its changes over the period in which participants reported changes in food intake. Moreover, the study was conducted in a group representing the same cultural background, so future studies should be expanded to other cultural backgrounds. Finally, the study did not allow for an in-depth analysis of the identified homogeneous groups (clusters) in terms of the motives for the changes made, nor the relationship between changes in food intake and the magnitude of changes in diet quality. Therefore, further research could focus on individual clusters to understand what triggered the changes.

## 8. Practical and Theoretical Implications

The results show that practitioners, i.e., educators and dietitians, should motivate people to change their diet by encouraging them to simultaneously introduce changes in two directions, i.e., to increase the consumption of health-beneficial products and reduce the consumption of unfavorable ones. Making even slow two-way changes is conducive to improving diet quality, while no change is associated with a worse diet. Moreover, our change-based segmentation analysis provides insight into previous changes that can be incorporated into the design and implementation of sustainable and healthy food policies. Previous changes have been shown to support their continuation, which can impact consumer decision-making, including information seeking, beliefs, attitudes and intentions, and behavioral outcomes. In the future, special attention should be paid to consumers who currently do not change their diet and whose diet is of relatively poor quality. Therefore, research on the role of social norms and social influences in dietary change is needed, especially concerning meat consumption. Furthermore, identifying the potential and effectiveness of policy measures to shift food production and consumption towards plant-based diets should be a challenge. Therefore, the development and implementation of measures to influence dietary patterns and food choices (e.g., through dietary guidelines, education, and campaigns) is expected. Furthermore, the market environment (e.g., taxation, agricultural subsidies, and regulations) should be considered important in transitioning towards a more plant-based diet.

The study’s findings may challenge researchers to understand how the previous experiences of changing eating habits, such as limiting meat intake, interact with subsequent changes, including those involving the same behavior. Scientific evidence on behavior-change experiences is significantly underrepresented compared to the domain of motivation when explaining eating behavior. The use of experiences with changes in food intake can make an essential contribution to explaining both the intention to make a change and increasing the effectiveness of the change by tailoring strategies to individual characteristics.

## Figures and Tables

**Table 1:** Characteristics of the study sample.

Variables	N * = 1003	Total Sample %
Gender		
Men	483	48.2
Female	520	51.8
Age (years)		
18–24	104	10.4
25–34	193	19.2
35–44	205	20.4
45–54	162	16.2
55–64	221	22.0
65 and above	118	11.8
Education		
Primary	100	10.0
Vocational	180	17.9
Secondary	403	40.2
Higher	320	31.9
Place of residence		
A village	377	37.6
A town with less than 20,000 inhabitants	139	13.9
A city with 20,000–99,999 inhabitants	187	18.6
A city with 100,000–199,999 inhabitants	104	10.4
A city with 200,000–499,999 inhabitants	93	9.3
A city with 500,000+ inhabitants	103	10.3
Financial situation		
There is enough money for everything without saving	170	16.9
We live frugally and have enough money for everything	377	37.6
We live very frugally to save for major purchases	269	26.8
There is only enough money for the cheapest food and clothing	88	8.8
There is only enough money for the cheapest food, not enough for clothing	41	4.1
There is not enough money even for the cheapest food and clothing	13	1.3
It is difficult to say	45	4.5
Age (mean; SD in years) (range)	45.4; 15.55 (18–83)	

* N—number of participants.

**Table 2:** Changes in food consumption during the last 2 years (N = 1003).

Food Products	I Have Not Consumed % (N)	Consumption		
		Decreased % (N)	Did Not Change % (N)	Increased % (N)
So-called red meat	5.5 (55)	42.5 (426)	48.0 (481)	4.1 (41)
So-called white meat	1.9 (19)	22.7 (228)	63.5 (637)	11.9 (119)
Milk and dairy products	3.7 (37)	19.0 (191)	63.2 (634)	14.1 (141)
Legumes, e.g., beans, peas, soybeans, lentils	6.6 (66)	19.8 (199)	60.9 (611)	12.7 (127)

**Table 3:** Characteristics of the identified clusters according to sociodemographic characteristics.

		Clusters			
	Total	No Change (*n* = 480)	All Products Limited (*n* = 196)	Changes in Meat (*n* = 184)	Less Red Meat, More Other Products (*n* = 143)
Total (%)	100.0	47.9	19.5	18.3	14.3
Gender (%; *p* < 0.001) *					
Men	48.2	56.9	40.3	37.0	44.1
Female	51.8	43.1	59.7	63.0	55.9
Education (%; *p* = 0.05) *					
Primary	10.0	11.3	8.2	8.7	9.8
Vocational	17.9	19.4	20.4	15.2	13.3
Secondary	40.2	40.3	31.6	42.9	47.5
Higher	31.9	29.0	39.8	33.2	29.4
Age (in years; mean; standard deviation)	45.4; 15.55	46.0 ^a^; 15.75	46.4 ^b^; 15.20	45.6 ^c^; 15.67	41.9 ^abc^; 15.55

* significance; Chi-square test; ^a,b,c^ the means with the same superscripts differ statistically at *p* < 0.05; Student’s *t*-test (t).

**Table 4:** Characteristics of the identified clusters according to diet quality indices (mean; standard deviation).

Diet Quality Indices	Total	Clusters			
		No Change (*n* = 480)	All Products Limited (*n* = 196)	Changes in Meat (*n* = 184)	Less Red Meat, More Other Products (*n* = 143)
nHDI *	19.2; 10.25	20.1 ^a^; 9.59	16.0 ^ab^; 9.04	18.4 ^abc^; 9.67	21.4 ^bc^; 13.27
pHDI **	23.1; 12.14	21.8 ^a^; 11.48	21.2 ^b^; 11.10	24.1 ^bc^; 12.29	28.9 ^abc^; 13.59
DQI ***	4.0; 11.85	1.7 ^a^; 10.56	5.2 ^ab^; 11.63	5.7 ^ac^; 12.60	7.6 ^ad^; 13.70

* Non-healthy Dietary Index; ** Prohealthy Dietary Index; *** Diet-Quality Index; ^a,b,c,d^ the means with the same superscripts differ statistically at *p* < 0.05; Student’s *t*-test (t).

**Table 5:** Odds ratios for the intention to eat more plant food and less meat and meat products in the coming year, based on diet quality indices for the overall sample and the identified clusters.

Total Sample/Clusters	Diet Quality Indices	Intention to Eat More Plant Food Next Year					Intention to Eat Less Meat and Meat Products Next Year				
		β *	e^β^ *	95CI *		*p*-Value **	β	e^β^	95CI		*p*-Value
Total sample	pHDI	0.210	1.23	1.16	1.31	<0.001	0.101	1.11	1.05	1.17	<0.001
	nHDI	−0.024	0.98	0.94	1.02	0.286	−0.105	0.90	0.86	0.95	<0.001
	DQI	0.049	1.05	1.04	1.06	<0.001	0.044	1.05	1.03	1.06	<0.001
“No change” cluster	pHDI	0.176	1.19	1.10	1.30	<0.001	0.098	1.10	1.01	1.21	0.029
	nHDI	0.034	1.04	0.97	1.11	0.321	−0.033	0.97	0.89	1.05	0.423
	DQI	0.033	1.03	1.02	1.05	<0.001	0.031	1.03	1.01	1.05	0.002
“All products limited” cluster	pHDI	0.239	1.27	1.08	1.49	0.003	0.161	1.18	1.02	1.36	0.031
	nHDI	−0.130	0.88	0.78	0.99	0.032	−0.043	0.96	0.85	1.08	0.472
	DQI	0.070	1.07	1.04	1.11	<0.001	0.036	1.04	1.01	1.07	0.001
“Changes in meat” cluster	pHDI	0.165	1.18	1.01	1.38	0.042	0.084	1.09	0.96	1.23	0.173
	nHDI	−0.047	0.95	0.84	1.08	0.457	−0.143	0.87	0.77	0.97	0.016
	DQI	0.040	1.04	1.01	1.07	0.011	0.041	1.04	1.02	1.07	0.002
“Less red meat, more other products” cluster	pHDI	0.219	1.24	1.01	1.53	0.041	0.024	1.03	0.91	1.16	0.699
	nHDI	−0.025	0.98	0.87	1.10	0.6762	−0.140	0.87	0.79	0.96	0.0065
	DQI	0.044	1.05	1.01	1.08	0.0158	0.043	1.04	1.02	1.07	0.0023

* OR—point estimate (β e^β^), 95% confidence intervals; ** significance level of the Wald’s test; pHDI—Prohealthy Dietary Index; nHDI—Non-healthy Dietary Index; DQI—Diet Quality Index.

## Data Availability

The data is not publicly available because the data have not yet been made available in ‘publicly available databases’. However, the data presented in the study are available on request from the corresponding author.
